# Evaluation of new endodontic tooth models in clinical education from the perspective of students and demonstrators

**DOI:** 10.1186/s12909-021-02848-9

**Published:** 2021-08-24

**Authors:** Sareh Said Yekta-Michael, Christoph Maria Färber, Alexander Heinzel

**Affiliations:** 1 Department of Orthodontics, RWTH Aachen University, Aachen, Germany; 2 Interdisciplinary Center for Clinical Research, RWTH Aachen University, Aachen, Germany; 3 Department for Operative Dentistry, Periodontology and Preventive Dentistry, RWTH Aachen University, Aachen, Germany; 4 Department of Nuclear Medicine, RWTH Aachen University, Aachen, Germany

**Keywords:** Artificial tooth model (RCT), Education, Endodontics, Feasibility study, Physical properties

## Abstract

**Background:**

The quality of root canal treatments performed by undergraduate students is often unsatisfactory questioning the current methods of teaching. Based on treatment errors made by students participating the endodontic courses at RWTH Aachen University (Germany), new radiopaque artificial root canal treatment models (DRSK RCT; incisor, premolar, molar) were designed and developed. The aim of the study was to evaluate these models by groups of students and demonstrators.

**Methods:**

A total number of 60 students and seven demonstrators from a single institution (RWTH Aachen) participated in this study. They performed endodontic treatments on either initial versions of the DRSK RCT or modified versions. The initial versions were evaluated by students (*n* = 25) and demonstrators (*n* = 7). The obtained questionnaire was conducted as 7-point Likert-Scale covering the topics material properties, feeling while performing exercises and perception of its closeness to reality via 19 items (students) and 21 items (demonstrators). According to the evaluations several alterations were applied to the DRSK RCT, the whole study was repeated and evaluated by different students (*n* = 35) and the same demonstrators (*n* = 7). Additionally, the demonstrators blindly evaluated the quality of root canal treatments performed by the students (*n* = 35) on the modified DRSK RCT. Comparisons between the initial versions and the modified versions were calculated using Chi-squared tests.

**Results:**

Students as well as demonstrators positively evaluated both variants of the DRSK RCT with especially high ratings in the overall evaluation. Students’ rating of the pulp anatomy significantly increased from 5.4 ± 1.1 (mean ± SD) to 5.9 ± 0.9 (mean ± SD; *p* < 0.05) for the modified model. Likewise, students felt that the ability to flare root canals improved after alterations have been applied. Ratings significantly increased from 4.8 ± 1.6 (mean ± SD) to 5.6 ± 1.0 (mean ± SD; *p* < 0.05).

**Conclusion:**

The results indicate that the DRSK RCT is a promising candidate to be used as an alternative to extracted teeth or as an additional tool for improving dental education. However, some limitations of our analysis have to be considered.

**Supplementary Information:**

The online version contains supplementary material available at 10.1186/s12909-021-02848-9.

## Background

Several studies point out that the quality of root canal treatments performed by undergraduate students is often poor [1–5] thereby questioning the quality of teaching endodontics [6]. The traditional preclinical training involved practicing the procedure on extracted natural teeth. However, this practice was fraught with concerns over infection control and required disinfection of the teeth [7]. Some materials traditionally used for the purpose of efficient disinfection, such as formalin, proved to have hazardous effects of their own [8]. Furthermore, the supply of natural teeth is not infinite, and combined with the dwindling number of extracted intact teeth - probably as a result of improvement in health standards - it presents a problem for instructors and students in preclinical endodontics [9].

The simplest of the artificial endodontic training models come in the shape of endodontic blocks with a built-in conduit that approximates in its shape and diameter the root canals of natural teeth [10]. Because they do not represent the external anatomy of the crown and root, these endodontic blocks are of limited educational value [11]. These models do not permit learning how to avoid the procedural problems related to the distance between the canal and external surface of the tooth and lateral or apical perforations of the roots [12].

Further advances in 3D printing technology promised more sophisticated models simulating a complete tooth including a hollow space representing the root canal system [13].. Although studies found artificial tooth models suitable for endodontic training [14], the results of these studies suggest that complete replacement of natural teeth with artificial teeth for endodontic training should be regarded with caution [15, 16]. To be suitable for the desired learning experience, the models are expected to feature physical properties as similar as possible to those of a natural tooth.

Finally, a recent study introduced the concept of 3D-printed replicas of extracted teeth for endodontic training [17]. In this study, the researchers produced exact copies of natural teeth and measured their properties and accuracy. However, there remain limitations associated with this concept [18]. Any modification of the model design, including shape, curvature, length, and width of the canals, falls beyond the scope of the proposed simple reproduction of a micro-computed tomography (micro-CT) file [18]. This can only be achieved by employing computer-aided design software applications, which - owing to their sophistication - normally necessitates enlisting the help of expert personnel with computer design skills [17].

The aim of the study was to develop and evaluate tooth models that closely resemble the real tooth anatomy in order to best prepare students for endodontic treatment of patients. To that end we designed artificial root canal treatment models (DRSK RCT) in two versions (initial version, modified version). Each version was evaluated by separate group of students as well as a group of demonstrators with regard to its material properties, feeling while performing exercises, and resemblance to properties of natural teeth using. We hypothesized an improvement of students’ and demonstrators’ evaluation of the modified version compared to the initial version of the models. The null hypothesis assumes no differences between the two versions.

## Methods

### Participants

**Fig. 1:**
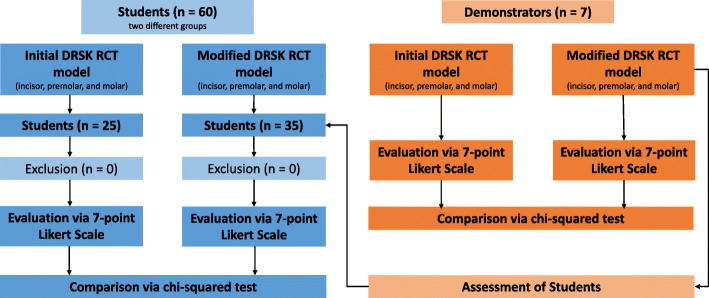
Illustration displaying the study design

The present study was conducted at the Department for Operative Dentistry, Periodontology and Preventive Dentistry of University Hospital RWTH Aachen in Germany. A total number of 60 students (part 1: *n* = 25; part 2: *n* = 35) and seven demonstrators participated in this study (Fig. 1).

All participating students had successfully passed the preclinical endodontic course (6th semester). The course comprises of endodontic exercises on extracted teeth and plastic blocks lectures and seminars as well as a written test and practical examination via an OSCE on extracted teeth. Two groups of students participated in different years. Each participant of the course volunteered to take part in the evaluation. The course was held by seven tutors who are fully qualified dentists with at least 2 years of experience in endodontics. The course lead had over 12 years of experience in endodontics.

### Development and evaluation of an artificial root canal treatment model (DRSK RCT)

We aimed to design two versions, firstly an initial version and then a modified DRSK RCT according to the evaluation of the initial version.

#### Initial DRSK RCT

**Fig. 2:**
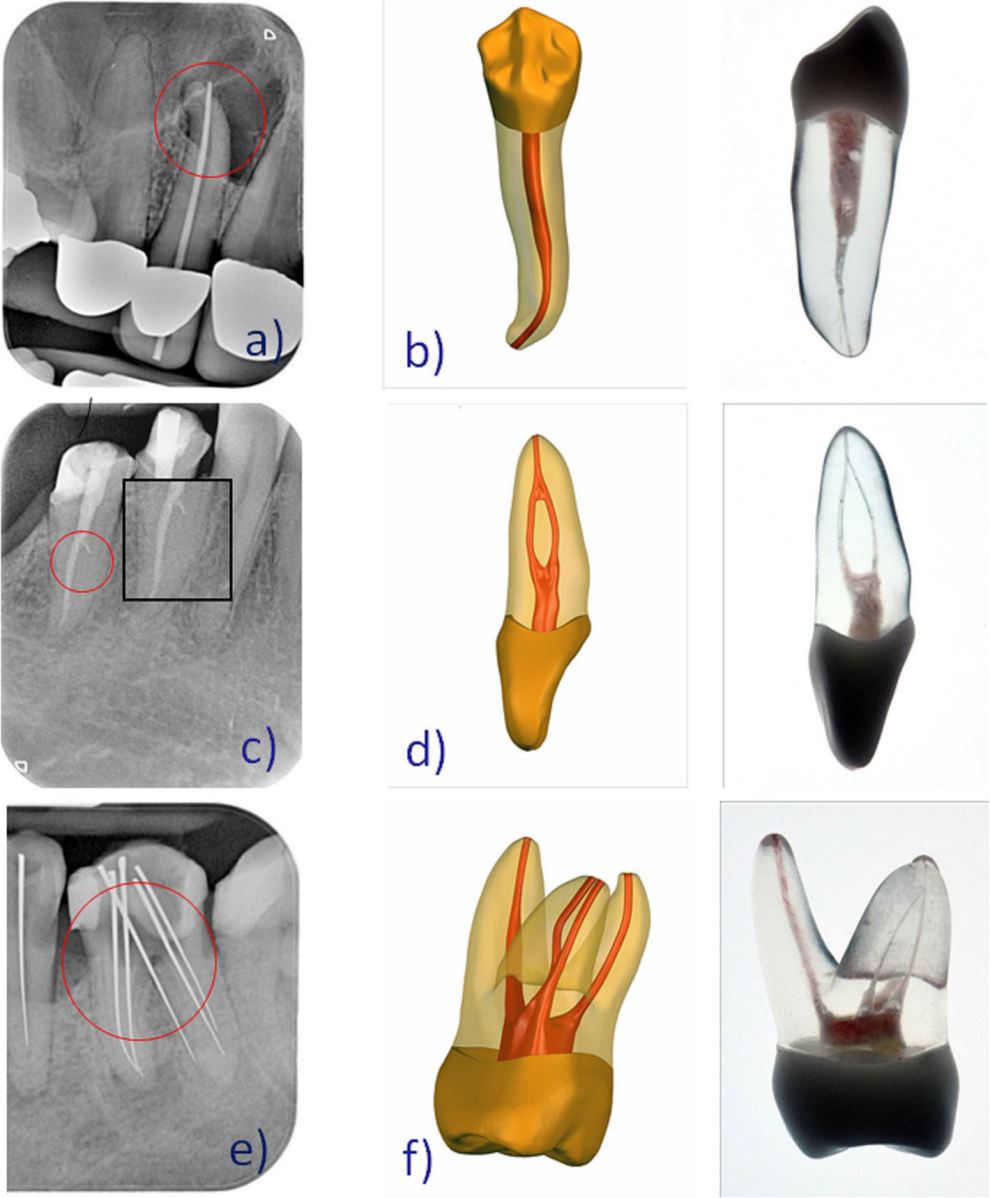
Radiographs showing typical treatment errors done in student courses (**a**, **c**, **e**) that led to the development of the initial model. Graphics and pictures of the modified endodontic tooth models (**b**, **d**, **f**). **a** Tooth 12 with j-shape root canal and a via falsa; **b** Premolar tooth model with an apically strongly curved root canal (draft + produced model); **c** Tooth 45 with a Type II shaped root canal and an incomplete obturated root canal; **d** Incisor tooth model with a Type II shaped root canal (draft + produced model); **e** Tooth 36 with a perforation of the pulp ground that was assumed to be a further root canal by students; **f** Molar tooth model with a hidden mb2 (draft + produced model)

Over a period of 5 years typical treatment errors of students in endodontic courses in RWTH Aachen University in Germany were assessed (examples are shown in Fig. 2 left column a, c, e). Based on these errors we designed a new artificial root canal treatment model, which was developed by DRSK Group AB, Sweden (https://drsk.com/pages/Training/RCT/root_canal_model_RCT.html). Tooth models were prepared in 3D with pulp cavity and root canal(s) that were considerably narrower in dimensions and shape than their natural counterparts. Tooth models contained complete and intact crowns and roots with a hollow space inside, simulating the pulp chamber and root canals. To achieve a more realistic representation, the hollow space in DRSK RCT was filled with a soft red-colored resin imitating the pulp. The models were completely transparent, which permitted the observation of the progression of the simulated treatment (Supplementary Figure 1).

We designed three tooth models, representing different types of teeth: incisors, premolars, and molars. The incisor was represented by a model of a first maxillary incisor. As an example of a premolar, a mandibular first premolar with a single root canal was chosen.

A maxillary first molar with three roots and four root canals was selected to demonstrate molars. The molar model featured a second mesiobuccal root canal (MB2) corresponding to the established predominance of this root canal configuration for maxillary first molars [19]. It should be noted that the models (initial and modified DRSK RCT) can be embedded in jar models (Supplementary Figure 2).

The initial version of DRSK RCT was evaluated by students (*n* = 25) and demonstrators (*n* = 7). The obtained questionnaire was conducted as 7-point Likert-Scale covering the topics material properties, feeling during performing exercises and perception of its closeness to reality via 19 items (students) and 21 items (demonstrators). Scale was from 1 (fully disagree) to 7 (fully agree) with 4 being “neither agree nor disagree”. All items were designed so that 1 represented the most negative score.

### Modified DRSK RCT

**Fig. 3:**
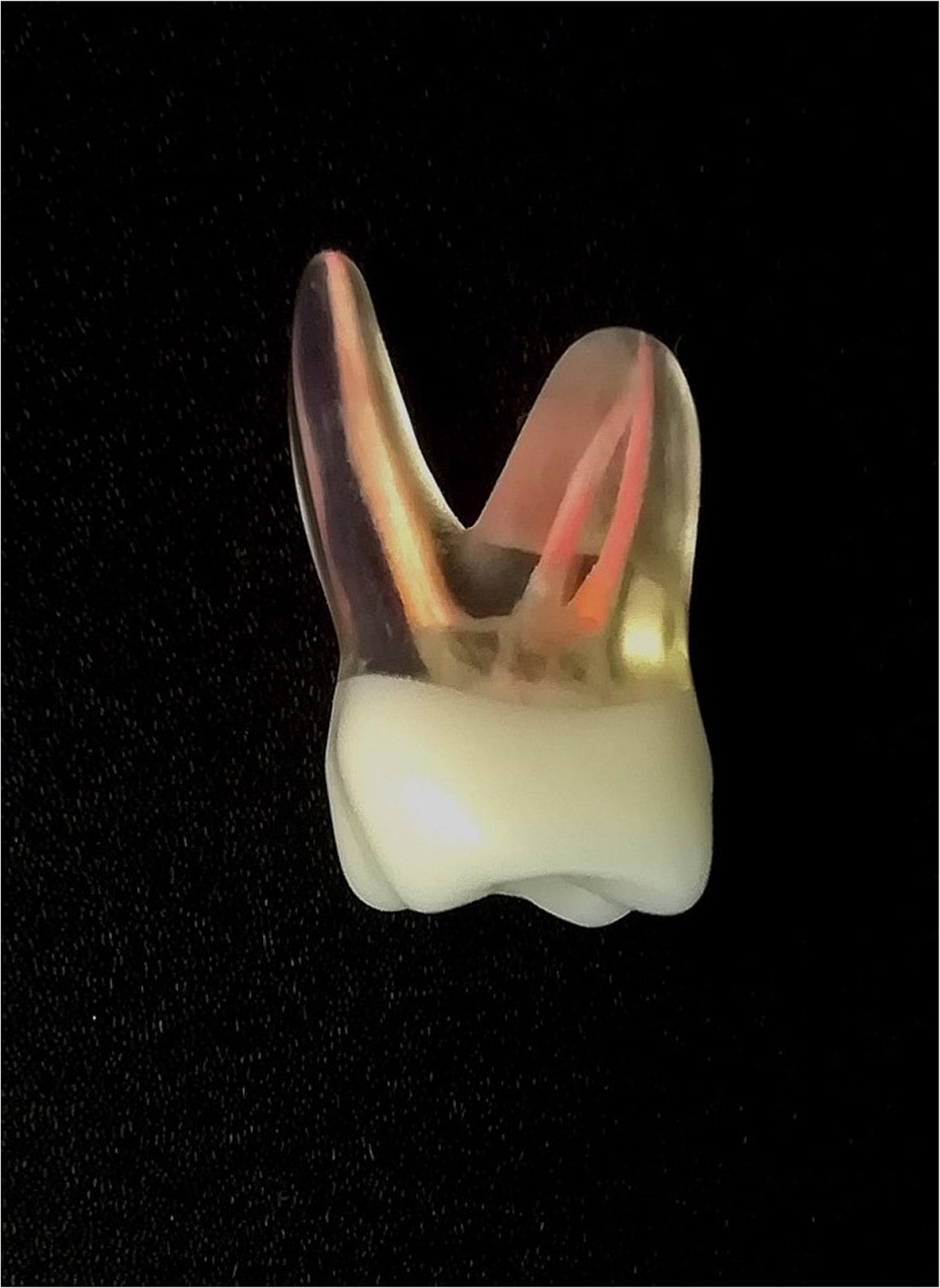
Picture of a modified DRSK RCT model of a maxillary first molar with completed root filling with gutta-percha points performed by a student with lateral condensation technique

According to the evaluations made by students and demonstrators several alterations were applied to the initial DRSK RCT. Hardness of material was increased to closer resemble the real life experience during the preparation process [20]. At the same time, root canals were further narrowed allowing sounding with C-pilot files matching size 06. The incisor received a second hidden root canal, which is one of the rarest configurations for this type of tooth. The location of the maxillary first molar’s second mesiobuccal root canal was altered in order to create a more realistic design. Finally, the transparent crown was whitened and thus, prevented the pulp chamber from the students’ direct view enabling students to practice trepanation of the pulp cavity (Fig. 3).

After modification another group of students (*n* = 35) and the same group of demonstrators (*n* = 7) evaluated the modified tooth models using the same questionnaires as for the initial models.

**Fig. 4:**
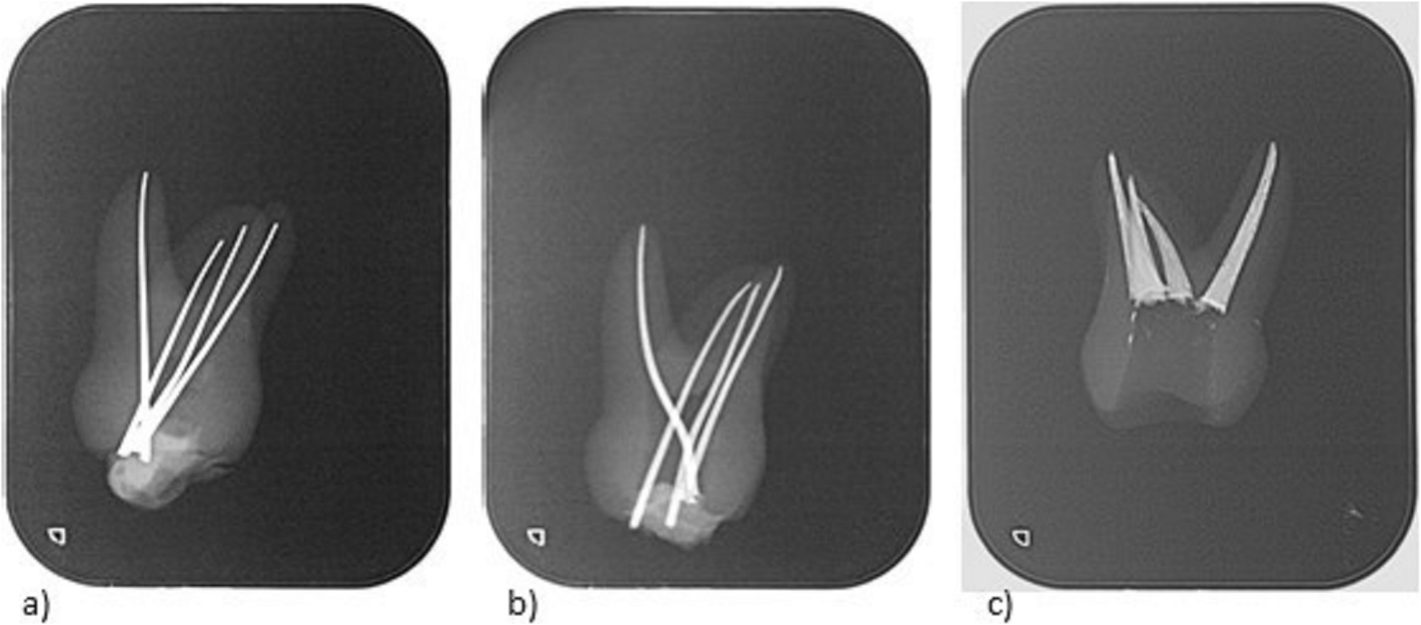
Radiographs of a maxillary first molar DRSK RCT tooth model: **a** lengths measuring with silverpoints, **b** control of gutta-percha master cones, **c** control of the completed root canal filling

Additionally, the demonstrators blindly evaluated the root canal treatments performed by the students (*n* = 35) on the modified DRSK RCT in order to assess its feasibility. A positive rating was given when all root canals were adequately treated with accurate lengths (0.5–1.0 mm before radiographic apex) and radiographically adequate density of root fillings (Fig. 4) [21]. The same group of demonstrators who evaluating the initial models, evaluated the modified model as well blindly assessed the students’ success of the second group. Outcome was considered as ‘negative’, when one canal was inadequately treated. The evaluation was done analogue to root canal treatments on real patients.

#### Endodontic training using DRSK RCT models

Before using DRSK RCT, all students practiced on extracted teeth and plastic blocks with simulated root canals. Then, the participants were asked to perform a routine root canal procedure on the DRSK RCT, starting with access cavity preparation using cylindrical diamond burs (Komet-Brassler, Lemgo, Germany). Therefore, a high-speed hand peace with water cooling was used. Access cavities were extended with Endo-Z burs (Dentsply Sirona, Bensheim, Germany). Preflaring was performed by using Mueller drills (Komet-Brasseler, Lemgo, Germany) and Gates-Glidden drills (Komet-Brasseler, Lemgo, Germany) in a crown-down technique. C-pilot files (VDW GmbH, Munich, Germany) were utilized for initial sounding of the root canals. After taking the radiograph (Fig. 4), hand files (K-Files, SybronEndo) were used for conventional root canal preparation.

Students flared the canals until size 35, that was set as the size of the master file. Students continued shaping the canals, using the step-back technique to enlarge them until size 60. Between change of instruments, canals were irrigated with 3% sodium hypochlorite solution, and patency was ensured by using size 10 files. Radiographs were taken during the process in accordance with the routine protocol (once to determine the working length, then with the master cone in place, and a post-operative radiograph). For taking radiographs, the setting of the X-ray machine was changed to levels normally used for children. Canal obturation in this study was performed using gutta-percha (ANTAEOS GuttaPercha Points, VDW GmbH, Munich, Germany) with AH26 as sealer in cold lateral condensation technique.

The demonstrators performed the same procedure of simulated root canal treatment - as described for the student group - on the DRSK RCT.

### Statistics

The accumulated data for the two sets of questionnaires of the students and the two sets of questionnaires of the demonstrators was entered into an Excel spreadsheet and means and standard deviations (SD) for each question were calculated. Data were analyzed using Chi-squared test or Fisher’s exact test as appropriate. Comparisons were performed for every single item obtained from students between the two groups: before and after modifications. In order to execute bivariate analysis, we collapsed the data into binary data. Participants answering 1–4 on Likert-scale were summarized to “disagree” and participants answering 5–7 were summarized as “agree” [22].

The significance of differences was determined at *p* < 0.05 for both tests. The evaluation of the students’ performance of root canal treatment on modified DRSK RCT tooth models was executed with descriptive statistics only. The software IBM® SPSS® Statistics 27.0 (IBM®, USA) was used for all statistical calculations. Graphics were created with Excel (Microsoft Office Excel 2007®).

## Results

For the root canal treatments on the endodontic tooth models, there were no drop outs in either the student or the instructor group in the first and second phase of the study.

The mean of the students’ ratings was 5.1 ± 0.4 (mean ± SD). After modifying the DRSK RCT, students’ mean rating increased to 5.5 ± 0.5 (mean ± SD). This was not significant.

**Fig. 5:**
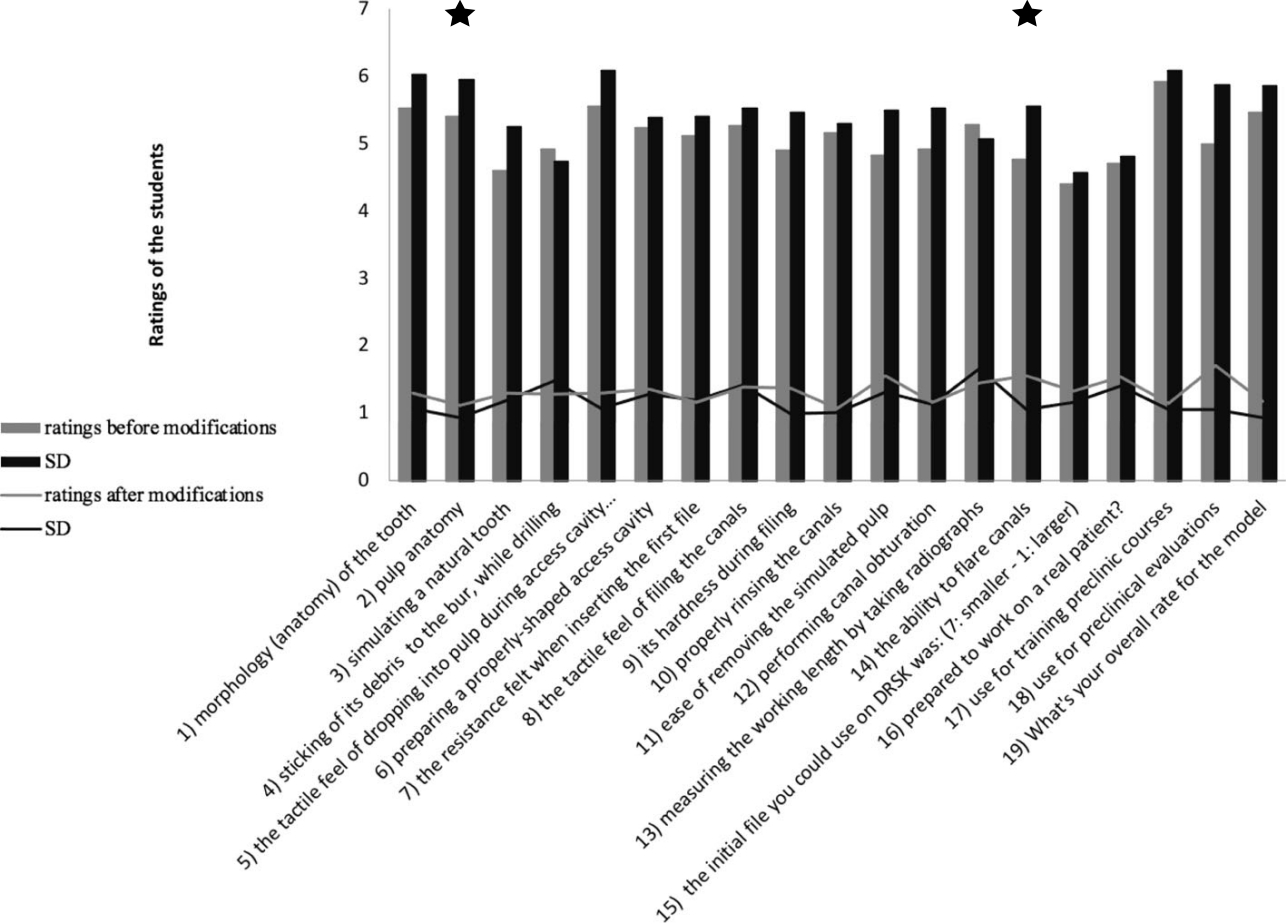
Bar chart showing the mean ratings of students on the tooth models in the initial model (grey) and the modified model (black). Line: standard deviation/ asterisk: *p* < 0.05

Students’ rating of the pulp anatomy significantly increased from 5.4 ± 1.1 (mean ± SD) to 5.9 ± 0.9 (mean ± SD; *p* < 0.05) for the modified model. Likewise, students felt that the ability to flare root canals improved after alterations have been applied. Ratings significantly increased from 4.8 ± 1.6 (mean ± SD) to 5.6 ± 1.0 (mean ± SD; *p* < 0.05) (Fig. 5).

**Fig. 6:**
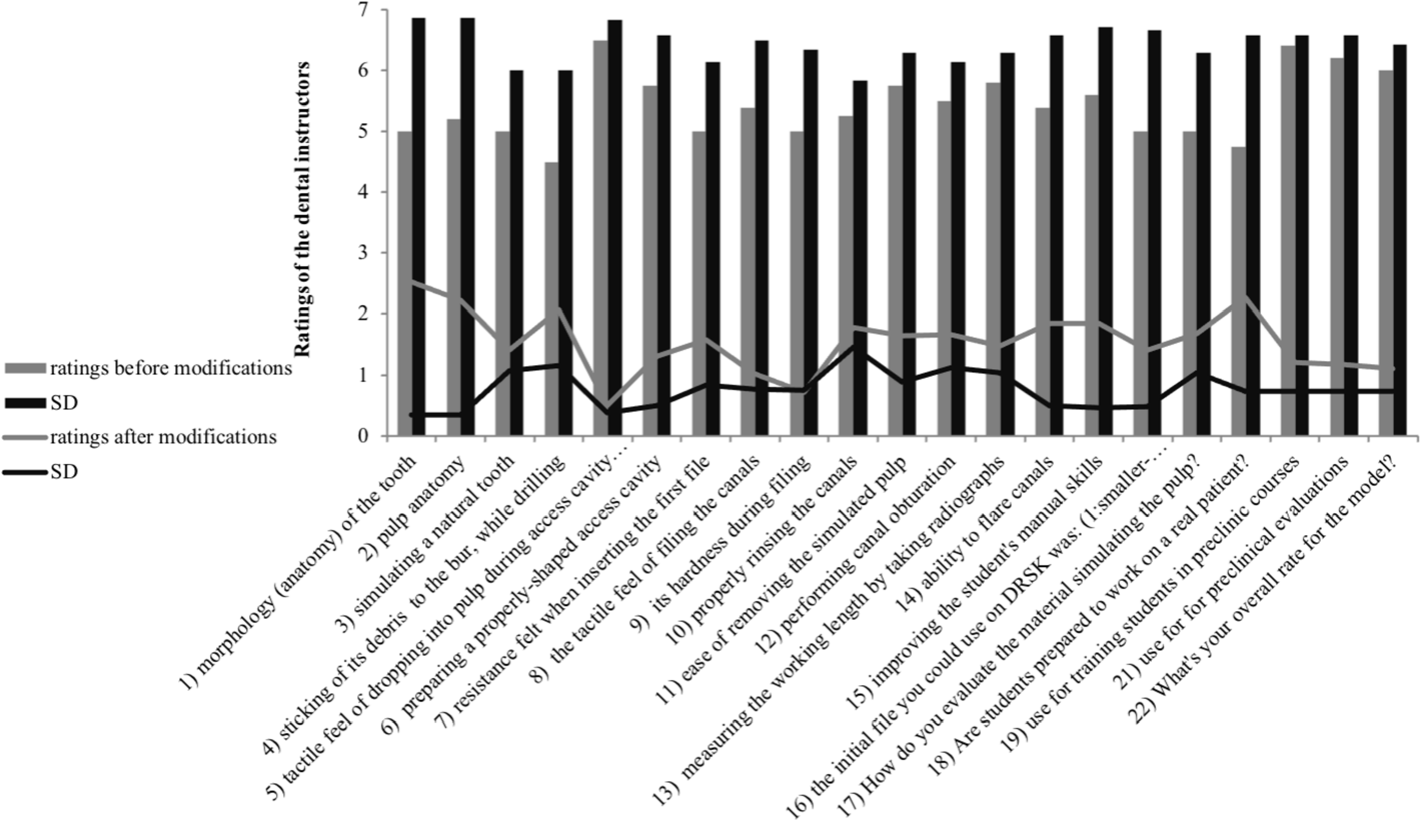
Bar chart showing the mean ratings of demonstrators on the tooth models in the initial model (grey) and the modified model (black). Line: standard deviation/ asterisk: *p* < 0.05

Demonstrators positively rated both tooth models. Mean of the demonstrators’ ratings non-significantly increased from 5.3 ± 1.5 (mean ± SD) to 6.2 ± 0.8 (mean ± SD) following model improvement (Fig. 6).

In the item-by-item comparison, the demonstrators’ rating did not show any significances, but almost, all ratings non-significantly increased in the second group (modified model; Table 1).

**Table 1:** Means and SD of students’ and demonstrators’ evaluation before and after modification were applied to the DRSK RCT

	Students before modifications (Mean ± SD)	Students after modifications (Mean ± SD)	Demonstrators before modifications (Mean ± SD)	Demonstrators after modifications (Mean ± SD)
Question 1	5.5 ± 1.3	6.0 ± 1.1	5.0 ± 2.8	6.9 ± 0.4
Question 2	**5.4 ± 1.1**	**5.9 ± 0.9**^**a**^	5.2 ± 2.5	6.9 ± 0.4
Question 3	4.6 ± 1.3	5.3 ± 1.2	5.0 ± 1.6	6.0 ± 1.2
Question 4	4.9 ± 1.3	4.8 ± 1.4	4.6 ± 2.1	6.0 ± 1.3
Question 5	5.6 ± 1.3	6.1 ± 1.1	6.5 ± 0.6	6.8 ± 0.4
Question 6	5.2 ± 1.4	5.4 ± 1.3	5.8 ± 1.5	6.6 ± 0.5
Question 7	5.1 ± 1.2	5.4 ± 1.2	5.0 ± 1.8	6.1 ± 0.9
Question 8	5.3 ± 1.4	5.5 ± 1.4	5.4 ± 1.1	6.5 ± 0.8
Question 9	4.9 ± 1.4	5.5 ± 1.0	5.0 ± 0.8	6.3 ± 0.8
Question 10	5.2 ± 1.1	5.3 ± 1.0	5.3 ± 2.1	5.8 ± 1.6
Question 11	4.8 ± 1.6	5.5 ± 1.3	5.8 ± 1.9	6.3 ± 1.0
Question 12	4.9 ± 1.2	5.5 ± 1.1	5.5 ± 1.9	6.1 ± 1.2
Question 13	5.3 ± 1.4	5.1 ± 1.7	5.8 ± 1.6	6.3 ± 1.1
Question 14	**4.8 ± 1.6**	**5.6 ± 1.0**^**b**^	5.4 ± 2.1	6.6 ± 0.5
Question 15	4.4 ± 1.3	4.6 ± 1.2	5.6 ± 2.1	6.7 ± 0.5
Question 16	4.7 ± 1.5	4.8 ± 1.4	5.0 ± 1.6	6.7 ± 0.5
Question 17	5.9 ± 1.1	6.1 ± 1.1	5.0 ± 1.9	6.3 ± 1.1
Question 18	5.0 ± 1.7	5.9 ± 1.1	4.8 ± 2.6	6.6 ± 0.8
Question 19	5.5 ± 1.2	5.9 ± 0.9	6.4 ± 1.3	6.6 ± 0.8
Question 20			6.2 ± 1.3	6.6 ± 0.8
Question 21			6.0 ± 1.2	6.4 ± 0.8

^a^ = *p* < 0.05 (Fisher’s exact test); ^b^ = *p* < 0.05 (Chi-squared test)

In light of the current COVID-19 pandemic, the demonstrators (*n* = 7) assessed the DRSK RCT as a possible substitute for patient treatments at 5.6 ± 0.5. Demonstrators (*n* = 7) positively evaluated 87.35% of the students’ (*n* = 35) performances on the modified training aids (Table 2).

**Table 2:** Assessment of root canal treatments performed by students on the DRSK RCT

Dental Instructor (*n* = 7)	Students’ Success Rate (*n* = 35)	Successful	Not Successful
Dental Instructor 1	88.57%	31	4
Dental Instructor 2	88.57%	31	4
Dental Instructor 3	85.71%	30	5
Dental Instructor 4	82.86%	29	6
Dental Instructor 5	88.57%	31	4
Dental Instructor 6	88.57%	31	4
Dental Instructor 7	88.57%	31	4

## Discussion

Students as well as demonstrators positively evaluated both variants of the artificial root canal treatment models with especially high ratings in the overall evaluation. Comparing the initial variant with the modified variant using chi-squared test a significant increase in the students’ evaluation was shown for the representation of pulp anatomy and the ability to flare canals.

Central aspects of the models comprise their ability to realistically simulate natural teeth and at the same time offer didactic properties that facilitate learning success. The simulation of natural tooth will be discussed by focusing on the evaluations of material properties, overall anatomical accuracy, and realistic representation of pulp. Moreover, we will discuss didactic aspects of the models such as translucent roots, radiopacity, standardized reproducible conditions in exams, as well as usability in distant learning (e.g. during a pandemic).

In order to provide a realistic learning experience the applied models should simulate the properties of natural tooth as closely as possible. When drilling an access cavity, the material must provide sufficient resistance such that its difference from the material filling the pulp chamber can be perceived clearly [16]. This aspect was already positively evaluated from both groups for the initial variant. However, compared to the other ratings there seemed to be potential for improvement. Therefore, one major alteration to the model after the first evaluation involved changing the material used in the manufacturing of DRSK RCT. Consequently, access drilling, root canal preparation, and obturation were performed more easily because the use of harder material offers more resistance. When entering the pulp chamber, the tactile feel is crucial for not harming the pulp floor by accident [16, 23]. This is important for beginners, as they are not used to paying attention to the difference between dentine and the hollow space of the pulp chamber. Sticky debris produced while shaping the root canals blocks the canals and cannot be irrigated easily. However, the stickiness of the debris is reduced when a harder material is used [20]. A material with hardness similar to that of dentine and cement of natural teeth accurately represents the tactile feel while performing root canal treatment on patients, which is the actual reason for using endodontic training aids. This is often perceived as a problem is other models [16, 17, 23].

With regard to anatomical accuracy the ratings were positive for both groups and models variants. The direct comparison of the two models showed increased ratings for both groups that were however not significantly different.

An important aspect represents the realistic representation of pulp. In root canal treatments on previous training models, endodontic files of size 15 were used for scouting. Owing to technical considerations, printing tooth models for endodontic training with optimal root canal diameter was challenging [17]. In a study that compared several endodontic training aids, a model named TrueTooth by DELabs, built on the basis of micro-CT scans of natural teeth, was favored owing to its anatomy and material properties being closer to reality [24]. For instance, diameters of the MB2 of TrueTooth #19 (02) are indicated to be 0.12 mm (apical part) and 0.28 mm (coronal part) on its manufacturer’s website. Because the initially used file for root canal treatment in real patient is often of a significantly lower size (6, 8, or 10), it is part of our department’s protocol of root canal treatment to use c-pilot files of size 6, 8, or 10 for scouting the root canal system. This needs to be practiced in preclinical training as well and thus requires models with very narrow root canals.

The 3D print technology used for producing the DRSK RCT made it possible to change the shapes of the root canals and modify them as desired without incurring substantial costs [25, 26]. The ratings by students and instructors alike point to the suitability of the studied model to be used in endodontic courses. This is reflected by positive ratings by an increase in the ratings of both groups (students and tutors) between the initial model compared to the modified one, however a significant increase was only shown for the students’ evaluation.

Moreover, the models should add didactic value to facilitate learning success. One important aspect is the translucence of the roots. The ambition to have models with translucent roots has been on record since as early as 1975 [27]. To that end, different methods have been devised to increase the transparency of the model by applying chemical agents. However, this is often accompanied by undesired effects such as altered physical properties [28, 29]. In the present study, the transparent roots of the model may also have influenced the students’ opinion about the DRSK RCT as a training aid in preclinical courses. Because endodontic treatments are performed inside root canals and obscured from view, students may often feel insecure when they are unable to see what they are doing. Making the treatment procedure visible could allow inexperienced students to gain a deeper understanding of the process [30]. If any error occurs, the cause can thus be quickly identified, which is essential for the students’ learning process [11]. Although transparent roots are described as beneficial for endodontic education [31], there may be drawbacks as reducing the treatment experience when the internal anatomy is visible. This could be encountered by using blocks of silicone or dental gypsum and by whitening the crown. Models with visible roots may also benefit research and testing of certain devices and equipment when it is required to have a direct view of the canals and observe how the equipment functions inside [29].

As radiopacity is the key to determine the correct working length [1, 3, 21], it is crucial that the apical third is clearly visible on radiographs, and thus the correct length of the gutta percha filling can be perceived [24]. Both model variants were radiopaque permitting the demonstrators to assess the success of students’ performance.

“Based on our experience of several years with 3D printed teeth three main benefits have to be mentioned. In the first place, 3D printed teeth are much easier available than natural teeth. In principle, any imaginable anatomical variant produced according to the needs for teaching. Also, the exact same variant can be reproduced multiple times to train or to perform an examination under standardized conditions. Finally, the time consuming act of collecting natural teeth for teaching and the risk of infection are no longer relevant [17].

After the introduction of the DRSK RCT the teaching in our endodontic course changed in various ways. Firstly, the visible roots permit the demonstrators to better explain the various steps of the root canal treatment as well as the evaluation of treatment success of students in addition to radiographic controls. Secondly, the models allow to specifically teach problematic anatomical aspects. For example, the models facilitate locating and flaring the MB2 of maxillary molars, one of the most frequently missed canal during endodontic treatment [32]. Finally, the option to have narrow root canals enables highly demanding training leading to a more realistic preparation for patient treatment.

Another advantage to use models is that they have the exact same anatomy and therefore create equal and fair conditions for every student [16, 17]. In contrast, there are no two natural teeth with identical anatomy [33]. Often, extracted teeth suffer from past treatments that make performing root canal treatments on them more complicated or even impossible. This could influence the feasibility to test endodontic treatment skills in an exam setting.

Moreover, during the COVID-19 pandemic it could be advantageous to incorporate this training aid into the clinical curriculum as supplying students with suitable patients to perform endodontic treatments becomes challenging [34]. Many patients currently avoid the student course out of fear of infection and postpone treatments [34]. In addition, as the number of cases increases, it is becoming more common for patients to be quarantined, making it impossible to carry out treatment properly. As many universities exclusively train students on phantom heads during the current pandemic the tooth model would perfectly fit into their curriculum [35]. Both, students and tutors rated the model as suitable for training and evaluation in preclinical courses and students also felt well prepared for their first root canal treatment on patients.

In order to ensure the usability of the modified variants the demonstrators evaluated root canal treatments of the second group of students on the modified models. The results indicate that the students were able to perform proper endodontic treatments on the modified tooth model. The students work on these models in the 7th semester 1 week before they have their first patient. In our experience, working on the customized DRSK RCT (including challenging less common anatomical variations) prepares the students to handle difficult cases and learn to manage unfamiliar situations before they are confronted with the challenges of real patients. Further studies are needed to prove the usefulness of the DRSK RCT by evaluating the outcome of treating the first patient after practicing on the DRSK RCT and comparing the results with those for a control group without prior experience of working on the DRSK RCT.

The following limitations have to be noted. Firstly, due to the small sample size, especially of the group of demonstrators our results may not be representative resulting in limited generalizability. To overcome this problem multicenter-studies are needed that combine the demonstrators from different faculties. Secondly, although almost all items showed improved ratings in the comparison of the initial variant and the modified variant, only two items differed significantly. This might be due to high initial ratings, so that further improvements assessed by a different group were difficult to measure.

Finally, the property of translucency facilitates teaching and learning on one hand, but on the other hand it does not represents the real treatment scenario. Thus, for advanced students the models have to be prepared to avoid translucency. This could be done by embedding the artificial tooth in a jar model compatible with commonly used phantom heads.

## Conclusion

Our data indicate that the DRSK RCT is a suitable candidate to be used as an alternative to extracted teeth or as an additional tool for improving dental education.

Important aspects of the models comprise their ability to realistically simulate natural teeth with regard to material properties, overall anatomical accuracy, and realistic representation of pulp. Moreover, it offers didactic properties that facilitate learning success including translucent roots, radiopacity, standardized reproducible conditions in exams, as well as usability in distant learning. However, some limitations of our analysis have to be considered.

## Supplementary Information

**Additional file 1: Supplementary figure 1.** Picture showing the initial version of the DRSK RCT model. a) graphic of the maxillary molar with translucent crown and roots. b) picture with occlusal view of the maxillary molar with the access cavity already established.

**Additional file 2: Supplementary figure 2.** Picture of mounted DRSK RCT model. The DRSK RCT model can be embedded in a jaw model to simulate a realistic clinical setting including rubber dam isolation for training and exam purposes.

## Data Availability

All data are available from the corresponding author on reasonable request.

## Abbreviations

COVID-19 pandemic: Coronavirus disease pandemic
DRSK RCT: Name of the tooth model presented in this study
MB2: Second mesiobuccal root canal
