# Supplementary figures and images for: Evaluation of new endodontic tooth models in clinical education from the perspective of students and demonstrators
Source: BMC Med Educ. 2021 Aug 24;21:447. doi: 10.1186/s12909-021-02848-9 (PMC8383916; doi:10.1186/s12909-021-02848-9)

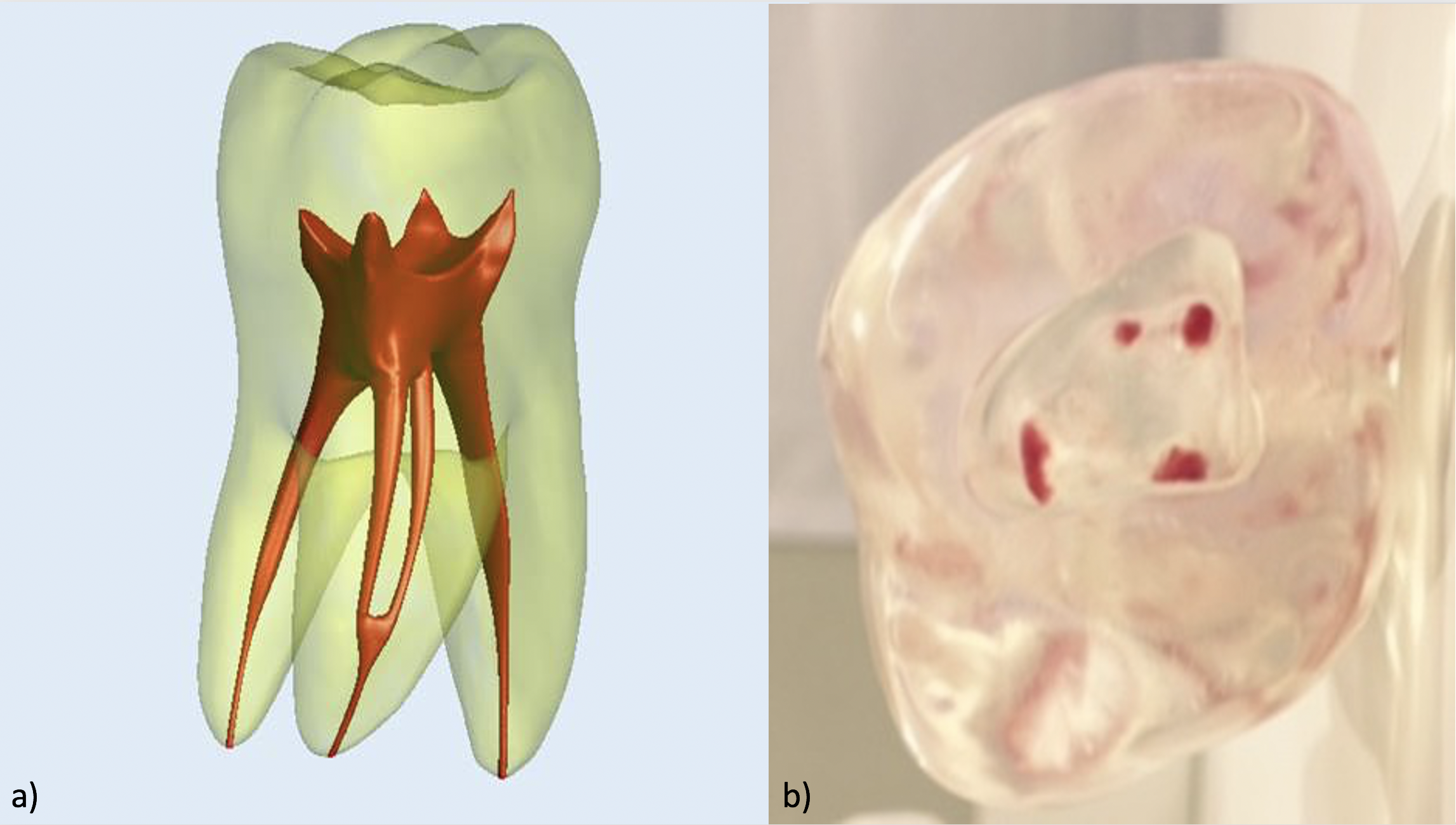

Supplement: Supplementary file 1 — Additional file 1: Supplementary figure 1. Picture showing the initial version of the DRSK RCT model. a) graphic of the maxillary molar with translucent crown and roots. b) picture with occlusal view of the maxillary molar with the access cavity already established. [file 12909_2021_2848_MOESM1_ESM.jpg]

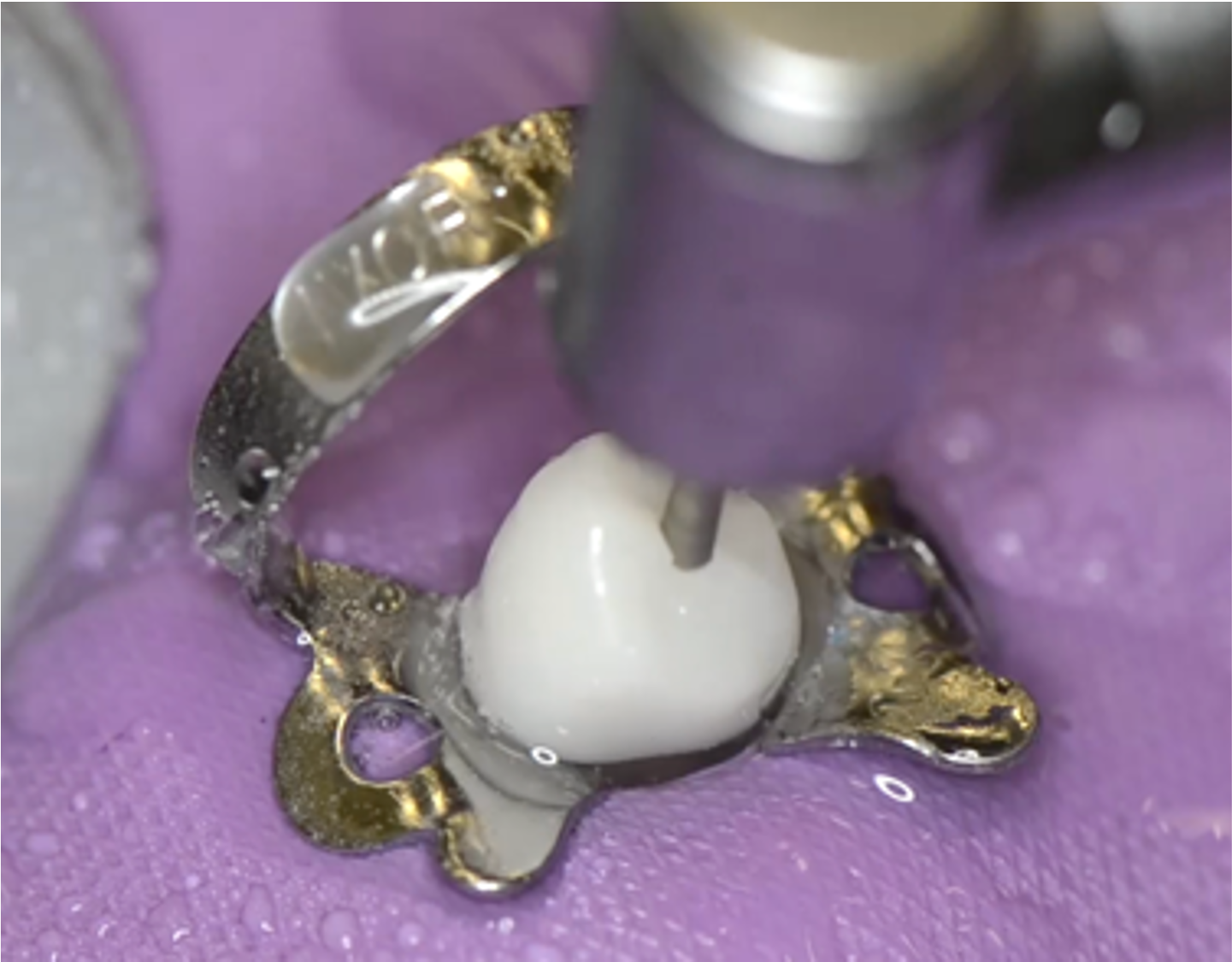

Supplement: Supplementary file 2 — Additional file 2: Supplementary figure 2. Picture of mounted DRSK RCT model. The DRSK RCT model can be embedded in a jaw model to simulate a realistic clinical setting including rubber dam isolation for training and exam purposes. [file 12909_2021_2848_MOESM2_ESM.jpg]
